# Astragaloside IV alleviates myocardial ischemia-reperfusion injury in rats through regulating PI3K/AKT/GSK-3β signaling pathways^1^

**DOI:** 10.1590/s0102-865020190070000008

**Published:** 2019-09-12

**Authors:** Dajun Wei, Hongjie Xu, Xiaodong Gai, Ying Jiang

**Affiliations:** I MD, Department of Cardiology, Affiliated Hospital, Beihua University, P.R. China. Technical procedures, acquisition of data, final approval.; II Master, Department of Oncology, Affiliated Hospital, Beihua University, P.R. China. Design of the study, critical revision, final approval.; III PhD, School of Medical Science, Beihua University, P.R. China. Statistics analysis, final approval.; IV Master, Health Service Center of Wenmiao Community, Changyi District, P.R. China. Manuscript writing, final approval.

**Keywords:** Myocardial Reperfusion Injury, Ischemia, Rats

## Abstract

**Purpose:**

To investigate the effect of astragaloside IV (As-IV) on myocardial ischemia-reperfusion (I/R) injury in rats and reltaed mechanisms.

**Methods:**

Sixty rats were randomly divided into sham-operated, control I/R and 2.5, 5 and 10 mg/kg As-IV groups, 12 rats in each group. The later three groups were intragastrically administered with As-IV for 7 days, with a dose of 2.5, 5 and 10 mg/kg, respectively. The myocardial I/R injury model was constructed in later four groups. At the end of reperfusion, the cardiac function indexes, serum lactate dehydrogenase (LDH) and creatine kinase (CK) levels, heart weight (HW)/body weight (BW) ratio and infarct size, and expressions of phosphatidylinositol-3 kinase/serine-threonine protein kinase (PI3K/AKT) and glycogen synthase kinase-3β (GSK-3β) proteins and the phosphorylated forms (p-AKT, p-GSK-3β) were determined.

**Results:**

Compared with control I/R group, in 5 and 10 mg/kg As-IV groups the left ventricular systolic pressure, fractional shortening and ejection fraction were increased, the left ventricular end-diastolic pressure was decreased, the serum LDH and CK levels were decreased, the HW/BW ratio and myocardial infarct size were decreased, and the p-Akt/Akt ratio and p-GSK-3β/GSK-3β ratio were increased (all P < 0.05).

**Conclusion:**

As-IV can alleviate the myocardial I/R injury in rats through regulating PI3K/AKT/GSK-3β signaling pathways.

## Introduction

**Figure 1:**
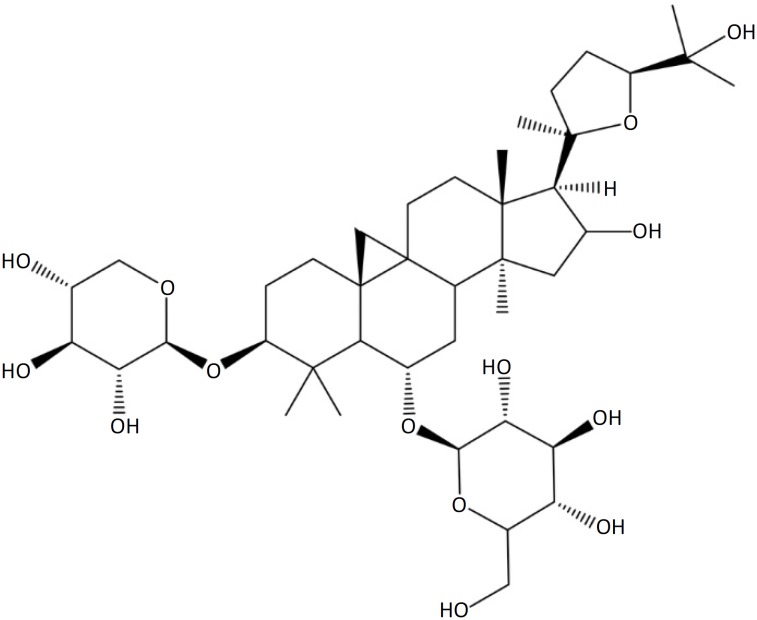
Structure of astragaloside IV.

Myocardial ischemia-reperfusion (I/R) injury is the lesion in which the blood perfusion is restored after the myocardial blood supply is interrupted for a certain period, leading to the injury or dysfunction in ischemic area^1^. Myocardial I/R injury can lead to the impairment of cardiac function and damage of myocardial cells, which increases the risk of cardiovascular events, such as myocardial infarction and arrhythmia, and seriously affects the prognosis of underlying diseases^2^ ^,^ ^3^. In addition, the myocardial I/R injury can limit the application of coronary thrombolysis treatment, interventional therapy and bypass surgery^4^. Therefore, reducing myocardial I/R injury is of great significance to improve the curative effect of cardiovascular diseases. Astragaloside IV (As-IV) is one of the important effective chemical constituents of *Astragalus membranaceus,* a widely used Chinese herbal medicine (Fig. 1).

The pharmacological effects of As-IV include immunity enhancement^5^, anti-inflammation^6^, anti-oxidation^7^, anti-virus^8^ and so on. In recent years, more and more attention has been paid to the effect of As-IV on cardiovascular system diseases. It is reported that As-IV can attenuate the viral myocarditis^9^, myocardial fibrosis^10^ and heart failure^11^. In addition, the previous studies have shown that As-IV has the protective effects on myocardial I/R injury, and the mechanisms are various^12^ ^–^ ^16^. Phosphatidylinositol-3 kinase/serine-threonine protein kinase (PI3K/AKT) and glycogen synthase kinase-3β (GSK-3β) are important signal transduction pathways in cells, which play important biological roles in cell apoptosis, survival and proliferation^17^. Study has shown that, the PI3K/AKT/GSK-3β signaling pathways are involved in the occurrence of myocardial I/R injury^18^. Therefore, the study was carried out to investigate the protective effect of As-IV on myocardial I/R injury in rats and the relations with PI3K/AKT/GSK-3β signaling pathways. The object was to provide a basis for further clarifying the mechanism for the protective effect of As-IV on myocardial I/R injury.

## Methods

This study was approved by the ethics committee of the Affiliated Hospital of Beihua University. All animal procedures followed the Principles of Laboratory Animal Care and were in accordance with the Guide for the Care and Use of Laboratory Animals by the National Institutes of Health.

Sixty healthy male Sprague Dawley rats (220-240g) were randomly divided into sham-operated, control I/R and 2.5, 5 and 10 mg/kg As-IV groups, 12 rats in each group. In 2.5, 5 and 10 mg/kg As-IV groups, the rats were intragastrically administered with As-IV, with a dose of 2.5, 5 and 10 mg/kg, respectively (different amount of As-IV was dissolved in 1% sodium carboxymethyl cellulose solution). In the control and control I/R groups, the rats were intragastrically administered with 1% sodium carboxymethyl cellulose solution. The administration was performed once a day, for 7 successive days.

### Construction of myocardial I/R injury control I/R

After 60 min from the last intragastrical administration, the myocardial I/R injury model was constructed in control I/R and 2.5, 5 and 10 mg/kg As-IV groups. The rats were intraperitoneally injected with 100 mg/kg sodium pentobarbital for anesthesia. The needle electrodes were inserted subcutaneously into the left forelimb, right forelimb and left hindlimb to continuously monitor the lead II electrocardiogram. The endotracheal intubation was performed. The thoracotomy was conducted from left 1-3 ribs, and the positive pressure ventilation was performed. The heart was exposed, and the pericardium was cut open. The left anterior descending coronary artery was ligated with 6-0 Prolene ligature for 30 min, and then the ligature was cut off for reperfusion for 120 min. The ST segment elevation and recovery of limb lead electrocardiogram presented the success of coronary artery disconnection and recanalization. The rats meeting the above criteria entered the later experiments. In the sham-operated group, only the ligature threading was performed, without ligatation, and the remaining steps were the same with other groups. After reperfusion, the rats were weighed.

### Measurement of cardiac function

The cardiac function of rats was measured at the end of reperfusion. The left ventricular systolic pressure (LVSP) and left ventricular end-diastolic pressure (LVEDP) were detected using the PowerLab Data Acquisition and Analysis System (ADInstruments, Australia). The fractional shortening (FS) and ejection fraction (EF) were detected using HD15 Color Doppler Ultrasound Diagnostic System (Phillips, Netherlands). The specific operations were carried out in accordance with the instructions of instruments

### Determination of serum lactate dehydrogenase and creatine kinase levels

After measurement of cardiac function, 5.0 ml of blood were immediately taken from the common carotid artery. After centrifugation at 2000 rpm for 15 min, the serum was obtained. The serum lactate dehydrogenase (LDH) and creatine kinase (CK) levels were determined by chemical colorimetry. The operation procedure was carried out according to the instructions of kits (Shanghai Lanpai Biotechnology Co., Ltd., Shanghai, China).

### Determination of heart weight/body weight ratio and infarct size

After blood taking, the rats were killed by excessive anesthesia. The heart was quickly removed, and weighed. The heart weight (HW)/body weight (BW) ratio was calculated. After washing with normal saline, the left anterior descending branch of coronary artery was ligated again and perfused with 1% Evans blue. Then, the left ventricle was cut into 4-5 slices, and re-stained with 2,3,5-triphenyltetrazolium chloride (TTC) for 15 min. The nonischemic zone (blue area), myocardium-at-risk (nonstained area by Evans blue), and infarct zone (nonstained area by TTC) were outlined and areas quantified digitally using Image J 1.46 analysis software (European Molecular Biology Laboratory Inc., Oxford, UK). The infarct size (%) was expressed as the ratio of infarct zone area to myocardium-at-risk area.

### Western blotting

The myocardial tissue was collected. The protein was extracted using modified RIPA buffer, and the concentration of protein was determined by BCA colorimetry. The protein was separated by 10% SDS-polyacrylamide gel electrophoresis and transferred to nitrocellulose filter membranes. The membranes were blocked with blocking solution at 37°C for 1.5 h, followed by washing with TBST for 3 times. The membranes were incubated with primary antibody (anti-Akt, anti-phosphorylated (p)-Akt, anti-GSK-3β, anti-p-GSK-3β, anti-β-actin) at 4°C overnight. After washing with TBST for 3 times, the membranes were incubated with horseradish peroxidase-labeled second antibody at 37°C for 1 h, followed by washing with blocking solution for 3 times and washing with TBST for 3 times. The membranes were stained, and imaged. The optical density of each brand was analyzed. The relative expression level of target protein was expressed as ratio of its optical density to that of β-actin. The ratios of p-Akt/ Akt and p-GSK-3β/GSK-3β presented the phosphorylation levels of PI3K/AKT and GSK-3β protein, respectively.

### Statistical analysis

The SPSS 20.0 software was used for statistical analysis. Data were presented as the mean±SD. Grouped data were analyzed using a one-way analysis of variance followed by the Student-Newman-Keuls test. A P < 0.05 was considered to be statistically significant.

## Results

### Mortality of rats

Sixty rats were used in this study. No rat dying in sham-operated group during the experiment. During the modeling, there were 1, 2, 2 and 1 rats dying in control I/R and 2.5, 5 and 10 mg/kg As-IV groups, respectively. The causes of death included tracheal intubation failure, ventricular fibrillation immediately after ligation and malignant arrhythmia.

### Comparison of cardiac function indexes among five groups

At the end of reperfusion, compared with sham-operated group, in control I/R group the LVSP, FS and EF were significantly decreased, respectively (P < 0.05), and the LVEDP was significantly increased (P < 0.05). Compared with control I/R group, in 5 mg/kg As-IV and 10 mg/kg As-IV groups, the LVSP, FS and EF were significantly increased, respectively (P < 0.05), and the LVEDP was significantly decreased (P < 0.05) (Table 1).

**Table 1:** Comparison of cardiac function indexes among five groups.

Group	n	LVSP (mmHg)	LVEDP (mmHg)	FS (%)	EF (%)
Sham-operated	12	119.3±13.53	7.72±1.21	42.73±6.63	77.76±12.74
Control I/R	11	85.44±9.31^a^	11.45±2.01^a^	21.83±3.33^a^	41.42±6.37^a^
2.5 mg/kg As-IV	10	89.17±8.15^a^	10.21±1.32^a^	24.26±2.18^a^	43.63±7.29^a^
5 mg/kg As-IV	10	99.92±11.48^a^ ^b^ ^c^	9.48±1.07^a^ ^b^	27.47±4.49^a^ ^b^	56.37±9.82^a^ ^b^ ^c^
10 mg/kg As-IV	11	106.94±12.42^a^ ^b^ ^c^	9.21±1.28^a^ ^b^	36.52±5.32^a^ ^b^ ^c^ ^d^	61.83±10.32^a^ ^b^ ^c^

a P < 0.05 compared with sham-operated group;

b P < 0.05 compared with control I/R group;

c P < 0.05 compared with 2.5 mg/kg As-IV group;

d P < 0.05 compared with 5 mg/kg As-IV group.

As-IV, astragaloside IV; I/R, ischemia-reperfusion; LVSP, left ventricular systolic pressure; LVEDP, left ventricular end-diastolic pressure; FS, fractional shortening; EF, ejection fraction.

### Comparison of serum LDH and CK levels among five groups

**Figure 2:**
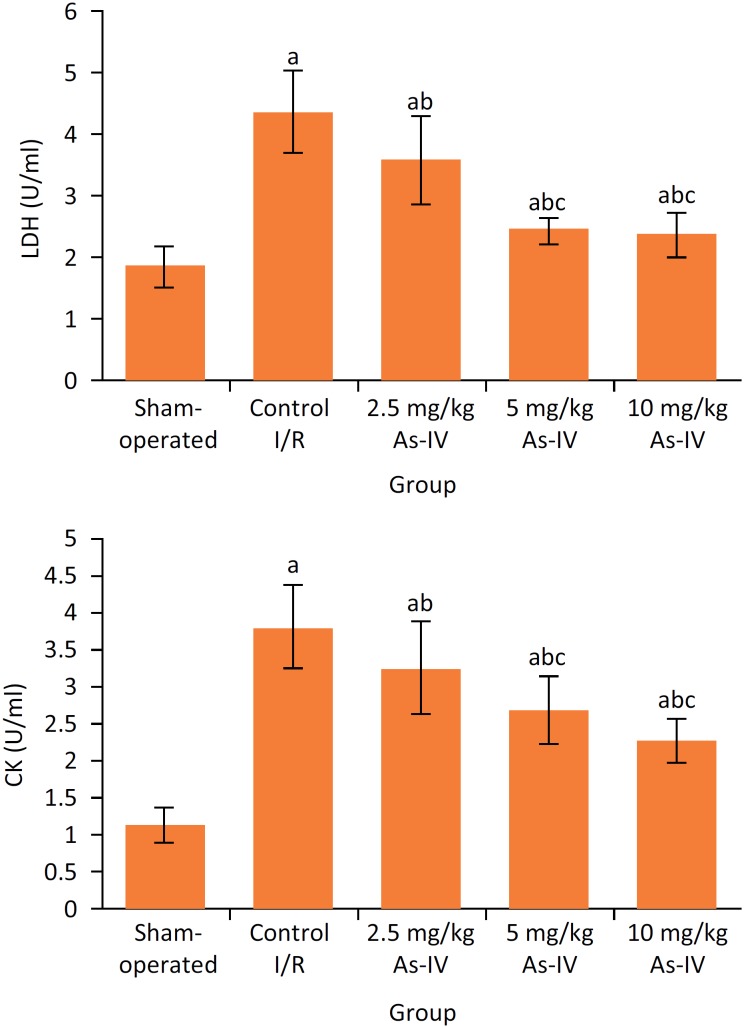
Comparison of serum LDH and CK levels among five groups. ^a^P < 0.05 compared with sham- operated group; ^b^P < 0.05 compared with control I/R group; ^c^P < 0.05 compared with 2.5 mg/kg As-IV group; ^d^P < 0.05 compared with 5 mg/kg As-IV group. As-IV, astragaloside IV; I/R, ischemia-reperfusion; LDH, lactate dehydrogenase; CK, creatine kinase.

As shown in Figure 2, after reperfusion, the serum LDH and CK levels in control I/R group were significantly higher than those in sham-operated group, respectively (P < 0.05). Compared with control I/R group, in 2.5 mg/kg As-IV, 5 mg/ kg As-IV and 10 mg/kg As-IV groups the serum LDH and CK levels were significantly decreased, respectively (P < 0.05).

### Comparison of HW/BW ratio and myocardial infarct size among five groups

**Figure 3:**
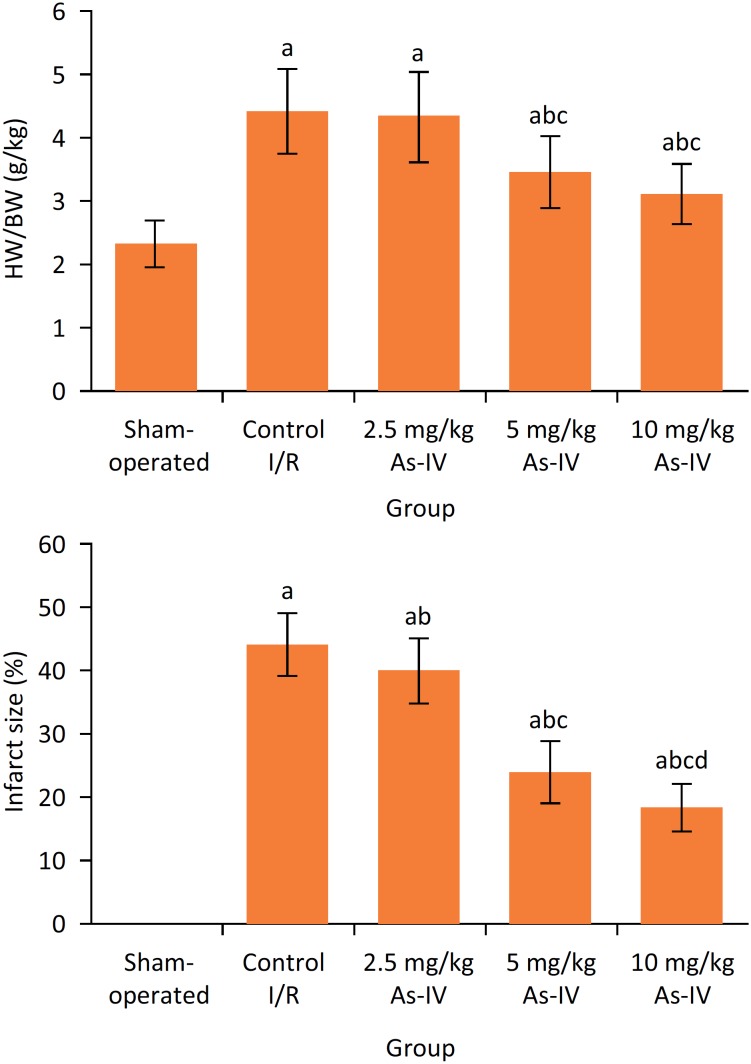
Comparison of HW/BW ratio and myocardial infarct size among five groups. aP < 0.05 compared with sham-operated group; bP < 0.05 compared with control I/R group; cP < 0.05 compared with 2.5 mg/kg As-IV group; dP < 0.05 compared with 5 mg/kg As-IV group. As-IV, astragaloside IV; I/R, ischemia-reperfusion; HW, heart weight; BW, body weight.

After reperfusion, compared with sham-operated group, in control I/R group the HW/BW ratio and myocardial infarct size were significantly increased, respectively (P < 0.05). Compared with control I/R group, the HW/BW ratio in 5 mg/kg As-IV and 10 mg/kg As-IV groups and myocardial infarct size in 2.5 mg/kg As-IV, 5 mg/kg As-IV and 10 mg/kg As-IV group were significantly decreased, respectively (P < 0.05) (Fig. 3).

### Comparison of phosphorylation levels of PI3K/AKT and GSK-3β protein in myocardial tissue

**Figure 4:**
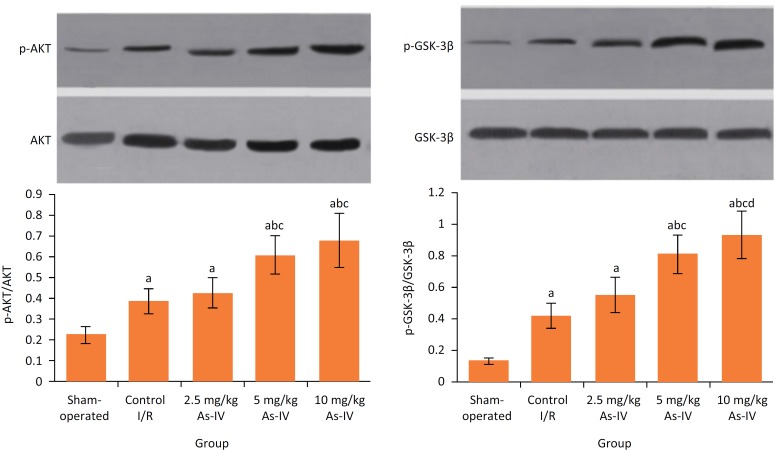
Comparison of phosphorylation levels of PI3K/AKT and GSK-3β protein in myocardial tissue. aP < 0.05 compared with sham-operated group; bP < 0.05 compared with control I/R group; cP < 0.05 compared with 2.5 mg/kg As-IV group; dP < 0.05 compared with 5 mg/kg As-IV group. As-IV, astragaloside IV; I/R, ischemia-reperfusion; AKT, serine-threonine protein kinase; p-AKT, phosphorylated serine-threonine protein kinase; GSK-3β, glycogen synthase kinase-3β; p-GSK-3β, phosphorylated glycogen synthase kinase-3β.

After reperfusion, compared with sham-operated group, in control I/R group the p-Akt/Akt ratio and p-GSK-3β/GSK-3β ratio were significantly increased, respectively (P < 0.05). Compared with control I/R group, the p-Akt/Akt ratio and p-GSK-3β/GSK-3β ratio in 5 mg/kg As-IV and 10 mg/kg As-IV groups were significantly increased, respectively (P < 0.05) (Fig. 4).

## Discussion

Restoration of reperfusion after myocardial ischemia within a certain period of time may be accompanied by I/R injury. The etiology of myocardial I/R injury is not yet clear, but it can reduce the cardiac function and increase myocardial cell damage^19^. Myocardial I/R injury is the main problem hindering the ischemic myocardium benefiting from therapy with reperfusion. *Astragalus membranaceus* is a widely used traditional Chinese medicine for the treatment of various heart diseases. It can improve the cardiac function and reduce cardiac myocyte apoptosis^20^. As-IV, the main active ingredient of *Astragalus membranaceus,* has a variety of biological activities, such as alleviating apoptosis of neurons around cerebral hemorrhage focus^21^ and attenuating myocardial infarction^22^, suggesting that it has a good cardiovascular protective effect. This study constructed the myocardial I/R injury model of rats and investigated the protective effect of As-IV. Result showed that, compared with control I/R group, in 5 mg/kg As-IV and 10 mg/kg As-IV groups, the LVSP, FS and EF were significantly increased, the LVEDP was significantly decreased, the serum LDH and CK levels were significantly decreased, and the HW/BW ratio and myocardial infarct size were significantly decreased. This indicates that, As-IV can alleviate the myocardial I/R injury in rats.

The P13K/AKT pathway is an important signal transduction pathway in the body, which plays an important role in cell survival, proliferation and apoptosis. When the myocardial I/R injury occurs, some kinases are activated and a series of cascade reactions are initiated to protect the myocardium^23^. It has been confirmed that the P13K/AKT signaling pathway plays a protective role in myocardial I/R injury control I/R of myocardial cell culture, ligation of coronary artery and perfusion of isolated heart^24^ ^–^ ^26^. Results of this study showed that, after reperfusion, compared with sham- operated group, in control I/R group the p-AKT/AKT ratio was significantly increased. This may be due to protective feedback of rats themselves when I/R injury occurs. Compared with control I/R group, the p-AKT/AKT ratio in 5 mg/kg As-IV and 10 mg/kg As-IV groups were significantly increased. This suggests that, As-IV can promote the phosphorylation of AKT, thus exerting the myocardial protective effects.

GSK-3 is a multifunctional serine/threonine protein kinase. There are two main subtypes of GSK-3 including GSK-3α and GSK-3β. GSK-3β is particularly closely related to the occurrence of apoptosis^27^. The regulation of GSK-3β is mainly realized by the phosphorylation and non-phosphorylation of GSK-3β and interaction with GSK-3β binding protein^28^. Many factors that regulate cell survival can phosphorylate GSK-3β through transduction of PI3K/AKT signal pathway, thus inhibiting its biological activity and ultimately promoting the cell survival^29^ ^,^ ^30^. Results of this study showed that, after reperfusion, compared with sham-operated group, in control I/R group the p-GSK-3β/GSK-3β ratio was significantly increased. This may be also caused by the protective feedback when I/R injury occurs. Compared with control I/R group, the p-GSK-3β/GSK-3β in 5 mg/kg As-IV and 10 mg/kg As-IV groups were significantly increased. This suggests that, As-IV can also promote the phosphorylation of GSK-3β, which is related to the myocardial protective effects.

## Conclusions

As-IV can alleviate the myocardial I/R injury in rats. The possible mechanism is related to it increasing phosphorylation of PI3K/AKT and GSK-3β protein and activating PI3K/AKT/GSK-3β signaling pathway. This study has provided a basis for further clarifying the mechanism for the protective effect of As-IV on myocardial I/R injury. In the next study, the upstream and downstream pathways of PI3K/AKT/GSK-3β signal pathways will be further studied to provide more abundant theoretical basis for clinical application of As-IV to prevention and treatment of myocardial I/R injury.

## References

[1] Turer AT, Hill JA (2010). Pathogenesis of myocardial ischemia-reperfusion injury and rationale for therapy. Am J Cardiol..

[2] Ekeløf SV, Halladin NL, Jensen SE, Zaremba T, Aarøe J, Kjærgaard B, Simonsen CW, Rosenberg J, Gögenur I (2016). Effects of intracoronary melatonin on ischemia-reperfusion injury in ST-elevation myocardial infarction. Heart Vessels..

[3] Ghiasi R, Mohammadi M, Majidinia M, Yousefi B, Ghiasi A, Badalzadeh R (2016). The effects of mebudipine on myocardial arrhythmia induced by ischemia-reperfusion injury in isolated rat heart. Cell Mol Biol (Noisy-le-grand)..

[4] Dominguezrodriguez A, Abreugonzalez P (2010). Myocardial ischemia-reperfusion injury: Possible role of melatonin. World J Cardiol..

[5] Zhang A, Zheng Y, Que Z, Zhang L, Lin S, Le V, Liu J, Tian J (2014). Astragaloside IV inhibits progression of lung cancer by mediating immune function of Tregs and CTLs by interfering with IDO. J Cancer Res Clin Oncol..

[6] Wang B, Chen MZ (2014). Astragaloside IV possesses antiarthritic effect by preventing interleukin 1β-induced joint inflammation and cartilage damage. Arch Pharm Res..

[7] Qiu LH, Xie XJ, Zhang BQ (2010). Astragaloside IV improves homocysteine-induced acute phase endothelial dysfunction via antioxidation. Biol Pharm Bull..

[8] Zhang Y, Zhu H, Huang C, Cui X, Gao Y, Huang Y, Gong W, Zhao Y, Guo S (2006). Astragaloside IV exerts antiviral effects against coxsackievirus B3 by upregulating interferon-gamma. J Cardiovasc Pharmacol..

[9] Gui J, Chen R, Xu W, Xiong S (2015). Remission of CVB3-induced myocarditis with Astragaloside IV treatment requires A20 (TNFAIP3) up-regulation. J Cell Mol Med..

[10] Chen P, Xie Y, Shen E, Li GG, Yu Y, Zhang CB, Yang Y, Zou Y, Ge J, Chen R, Chen H (2011). Astragaloside IV attenuates myocardial fibrosis by inhibiting TGF-β1 signaling in coxsackievirus B3-induced cardiomyopathy. Eur J Pharmacol..

[11] Dong Z, Zhao P, Xu M, Zhang C, Guo W, Chen H, Tian J, Wei H, Lu R, Cao T (2017). Astragaloside IV alleviates heart failure via activating PPARα to switch glycolysis to fatty acid β-oxidation. Sci Rep..

[12] Zhang WD, Chen H, Zhang C, Liu RH, Li HL, Chen HZ (2006). Astragaloside IV from Astragalus membranaceus shows cardioprotection during myocardial ischemia in vivo and in vitro. Planta Med..

[13] Tu L, Pan CS, Wei XH, Yan L, Liu YY, Fan JY, Mu HN, Li Q, Li L, Zhang Y, He K, Mao XW, Sun K, Wang CS, Yin CC, Han JY (2013). Astragaloside IV protects heart from ischemia and reperfusion injury via energy regulation mechanisms. Microcirculation..

[14] Si J, Wang N, Wang H, Xie J, Yang J, Yi H, Shi Z, Ma J, Wang W, Yang L, Yu S, Li J (2014). HIF-Ια signaling activation by post-ischemia treatment with astragaloside IV attenuates myocardial ischemia-reperfusion injury. PLoS One..

[15] Yu J, Zhang X, Zhang Y (2017). Astragaloside attenuates myocardial injury in a rat model of acute myocardial infarction by upregulating hypoxia inducible factor-1α and Notch1/Jagged1 signaling. Mol Med Rep..

[16] Zheng Q, Zhu JZ, Bao XY, Zhu PC, Tong Q, Huang YY, Zhang QH, Zhang KJ, Zheng GQ, Wang Y (2018). A Preclinical systematic review and meta-analysis of astragaloside IV for myocardial ischemia/reperfusion injury. Front Physiol..

[17] Chen X, Yan X, Guo L (2018). Inhibitory effect of Patrinia on BRL-3A cell apoptosis through the TLR4/PI3K/AKT/GSK3β and TLR4/P38/JNK signaling pathways. Mol Med Rep..

[18] Wang D, Zhang X, Li D, Hao W, Meng F, Wang B, Han J, Zheng Q (2017). Kaempferide protects against myocardial ischemia/reperfusion injury through activation of the PI3K/Akt/GSK-3β pathway. Mediators Inflamm..

[19] Wang X, Ha T, Hu Y, Lu Ch, Liu L, Zhang X, Kao R, Kalbfleisch J, Williams D, Li C (2016). MicroRNA-214 protects against hypoxia/reoxygenation induced cell damage and myocardial ischemia/reperfusion injury via suppression of PTEN and Bim1 expression. Oncotarget..

[20] Xu S, Xu B, Chen X, Zhu B, Wang Z (2004). Effect of Astragalus membranaceus on tumor necrosis factor-induced apoptosis in myocardial ischemia reperfusion injury in rats. Chin J New Drugs Clin Remedies..

[21] Qu YZ, Li M, Zhao YL, Zhao ZW, Wei XY, Liu JP, Gao L, Gao GD (2009). Astragaloside IV attenuates cerebral ischemia-reperfusion-induced increase in permeability of the blood-brain barrier in rats. Eur J Pharmacol..

[22] Cheng S, Yu P, Yang L, Shi H, He A, Chen H, Han J, Xie L, Chen J, Chen X (2016). Astragaloside IV enhances cardioprotection of remote ischemic conditioning after acute myocardial infarction in rats. Am J Transl Res..

[23] Wang Y, Zhang ZZ, Wu Y, Ke JJ, He XH, Wang YL (2013). Quercetin postconditioning attenuates myocardial ischemia/reperfusion injury in rats through the PI3K/Akt pathway. Braz J Med Biol Res..

[24] Tang J, Wang J, Kong X, Yang J, Guo L, Zheng F, Zhang L, Huang Y, Wan Y (2009). Vascular endothelial growth factor promotes cardiac stem cell migration via the PI3K/Akt pathway. Exp Cell Res..

[25] Han G, Song J, Ren J, Chen H (2011). Dual activation of AKT kinase and STAT-3 kinase is involved in the cardioprotection by remote limb ischemic postconditionin in rats. Heart..

[26] Liu H T, Wang H C, Guo W Y, Gao F (2007). Insulin protects the isolated heart from MI/R injury: a cross-talk between PI3K/Akt and SAPKs/JNKs. J Mol Cell Cardiol..

[27] Mokhtari B, Badalzadeh R, Alihemmati A, Mohammadi M (2015). Phosphorylation of GSK-3β and reduction of apoptosis as targets of troxerutin effect on reperfusion injury of diabetic myocardium. Eur J Pharmacol..

[28] Ren XM, Zuo GF, Wu W, Luo J, Ye P, Chen SL, Hu ZY (2016). GSK-3β-PP2Ac-NF-kB signaling axis. PLoS One..

[29] Zhang X, Shi M, Ye R, Wang W, Liu X, Zhang G, Han J, Zhang Y, Wang B, Zhao J, Hui J, Xiong L, Zhao G (2014). Ginsenoside Rd attenuates tau protein phosphorylation via the PI3K/AKT/GSK-3β pathway after transient forebrain ischemia. Neurochem Res..

[30] Park SJ, Jin ML, An HK, Kim KS, Ko MJ, Kim CM, Choi YW, Lee YC (2015). Emodin induces neurite outgrowth through PI3K/Akt/GSK-3β-mediated signaling pathways in Neuro2a cells. Neurosci Lett..

